# Purification and Characterization of a Thermostable *β*-Mannanase from* Bacillus subtilis* BE-91: Potential Application in Inflammatory Diseases

**DOI:** 10.1155/2016/6380147

**Published:** 2016-10-27

**Authors:** Lifeng Cheng, Shengwen Duan, Xiangyuan Feng, Ke Zheng, Qi Yang, Zhengchu Liu

**Affiliations:** Institute of Bast Fiber Crops, Chinese Academy of Agricultural Sciences, Changsha, Hunan 410205, China

## Abstract

*β*-mannanase has shown compelling biological functions because of its regulatory roles in metabolism, inflammation, and oxidation. This study separated and purified the *β*-mannanase from* Bacillus subtilis* BE-91, which is a powerful hemicellulose-degrading bacterium using a “two-step” method comprising ultrafiltration and gel chromatography. The purified *β*-mannanase (about 28.2 kDa) showed high specific activity (79, 859.2 IU/mg). The optimum temperature and pH were 65°C and 6.0, respectively. Moreover, the enzyme was highly stable at temperatures up to 70°C and pH 4.5–7.0. The *β*-mannanase activity was significantly enhanced in the presence of Mn^2+^, Cu^2+^, Zn^2+^, Ca^2+^, Mg^2+^, and Al^3+^ and strongly inhibited by Ba^2+^ and Pb^2+^. *K*_*m*_ and *V*_max_ values for locust bean gum were 7.14 mg/mL and 107.5 *μ*mol/min/mL versus 1.749 mg/mL and 33.45 *µ*mol/min/mL for Konjac glucomannan, respectively. Therefore, *β*-mannanase purified by this work shows stability at high temperatures and in weakly acidic or neutral environments. Based on such data, the *β*-mannanase will have potential applications as a dietary supplement in treatment of inflammatory processes.

## 1. Introduction

Mannan consists of a series of complex polysaccharides, which are found in the cell wall of marine algae [1]. The backbone is comprised of *β*-1,4-linked mannose residues. Konjac glucomannan is a randomly arranged polymer of *β*-1,4-linked glucose and mannose residues at ratio of 1.0 : 1.6. Both the backbones of mannan and Konjac are modified by *α*-1,6-linked galactosyl residues to form galactomannan and galactoglucomannan, respectively [2].

*β*-mannanase (EC 3.2.1.78) is a hemicellulase that attacks the internal glycosidic bonds of mannan backbone to release the condensed *β*-1,4-manno-oligosaccharides [3]. *β*-mannanases are widely applied in pulp and paper processing [4], feed [5], food [6], pharmaceutical [7], oil, and textile industries [8] to randomly hydrolyze the *β*-1,4 mannopyranoside linkage in mannan, galactomannan, glucomannan, and galactoglucomannan.

*β*-mannanase is widely produced by bacteria [9, 10], actinomycetes [11], fungi [12], plants, and animals [13]. Among them, *β*-mannanase from bacteria is wildely used because of numerous advantages, including extracellular secretion, economic production and purification, and novel characteristics, such as tolerance to heat and alkaline conditions [14].

Although multiple *β*-mannanase-producing bacteria have been reported [15, 16], they are far from the diverse industry needs. Currently, acidic and alkaline *β*-mannanase has been proposed to meet the industrial demands [17]. However, the requirements of high energy in production and the environmental impact limit their development. Neutral and weakly acidic *β*-mannanase with lower energy for production has attracted considerable interest over the past few years; however, it has rarely been characterized. It is clarified that *β*-mannanase with high activity in short fermentation time confers lower costs during the production procedures. Therefore, the exploitation of strains producing high *β*-mannanases activity is valuable and profitable. In current study, we isolated and preserved a powerful hemicellulose-degrading bacterium (BE-91). Then we explored the efficient purification process and characterized the enzymatic properties of its *β*-mannanase.

## 2. Materials and Methods

### 2.1. Microorganism, Media, and Fermentation Conditions

*B. subtilis* BE-91, a strain used for herbaceous fiber extraction, was identified and preserved by the Institute of Bast Fiber Crops, Chinese Academy of Agricultural Science (Changsha, Hunan, China).* B. subtilis* BE-91 was cultured in Petri dish containing 0.5% yeast extract, 1% NaCl, 0.5% Konjac glucomannan, 1% bacto tryptone, 0.05% trypan blue, and 1.5% agar. The seed medium was mainly composed of 0.1% glucose, 0.4% Konjac glucomannan, 0.3% beef extract, 0.2% yeast extract, 0.5% peptone, and 0.5% NaCl. The fermentation medium primarily consisted of 0.2% yeast extract, 0.7% Konjac glucomannan, 0.5% peptone, 0.3% beef extract, and 0.5% NaCl.* B. subtilis* BE-91 was first activated in the seed medium at 35 ± 1°C for 5.5 h. Subsequently, the suspension was serially diluted, spread onto Petri dishes, and incubated at 35 ± 1°C for 18 h. The single colony exhibiting the largest hydrolytic halo was transferred into an Erlenmeyer flask with the seed medium and cultured at 35 ± 1°C for 6 h at 180 rpm. Consequently, 2% culture was inoculated in the fermentation medium and cultured for 6 h at 35 ± 1°C at 180 rpm [18].

### 2.2. Classification of Strain BE-91

The 16S rDNA of strain BE-91 was PCR amplified from genomic DNA using the Bacterial Identification PCR Kit (TaKaRa, Japan). The obtained 16S rDNA was sequenced by the ABI 3730XL 96-capillary DNA analyzer. The primers were as follows: P1 5′-AGAGTTTGATCMTGGCTCAG-3′ and P2 5′-TACGGYTACCTTGTTACGACTT-3′. The resulting sequence aligned closely with the related standard sequences of other bacteria retrieved from GenBank. Neighbor-joining clusters were constructed by Mega 6.0 [19].

### 2.3. Enzymatic Assays

*β*-mannanase activity was estimated by initiating the reaction at 65°C for 10 min in 0.05 mol/L citric acid/0.1 mol/L Na_2_HPO_4_ buffer (pH 6.0) with 0.2% (w/v) Konjac glucomannan as substrate. The amounts of reducing sugar in the reaction were quantified based on a standard curve generated with mannose using the 3,5-dinitrosalicylic acid (DNS) method. One unit (IU) of *β*-mannanase activity was defined as the amount of protein producing 1 *μ*mol/L of reducing sugar per minute (e.g., mannose) under standard conditions [20].

### 2.4. Purification of *β*-Mannanase

The bacterial *β*-mannanase was purified using a two-step process involving ultrafiltration (Sartorius, Germany) and gel filtration. The fermentation liquid was fractionated orderly by 100 kDa, 50 kDa, and 5 kDa membrane thresholds. The solution filtered with 5 kDa < MW < 50 kDa was further purified on a Sephadex G-100 gel column (Ф1.6 cm × 100 cm, Pharmacia). The eluate was obtained at a rate of 0.5 mL/min and collected in 5 mL fractions. *β*-mannanase activity was determined by the DNS method, whereas the protein was quantified by the Coomassie brilliant blue staining against bovine serum albumin (BSA) standard [21].

### 2.5. The Determination of Apparent Molecular Weight

The molecular mass of the *β*-mannanase was determined by SDS-PAGE (Bio-Rad, USA), with 3% stacking gel and 12% separating gel [22]. The protein bands were stained with 0.01% Coomassie brilliant blue R-250 and destained with a water-methanol-acetic acid (9 : 9 : 2) solvent. Zymogram analysis was performed by the method of Chanhan [17]. The molecular weight of *β*-mannanase was derived from the relative mobility of molecular weight markers resolved simultaneously.

### 2.6. The Effect of Temperature on the Activity and Stability of *β*-Mannanase

The activity of *β*-mannanase was assayed at a range of temperatures between 50 and 70°C in 0.05 mol/L citric acid-0.1 mol/L Na_2_HPO_4_ buffer at pH 6.0. The thermostability was assessed by preincubating the enzyme, without a substrate, at different temperatures varying over 20–80°C for 30 min. The residual activity was promptly measured by the DNS method. The *β*-mannanase activity was considered to be 100% when preincubated at 4°C.

### 2.7. The Effect of pH on the Activity and Stability of *β*-Mannanase

*β*-mannanase activity was evaluated by incubating the purified enzyme at different pH conditions ranging from 4.0 to 8.0 in 0.05 mol/L citric acid-0.1 mol/L Na_2_HPO_4_ buffer at 4°C. The stability at a particular pH was tested by preincubating the purified enzyme, without a substrate, for 30 min in various 0.05 mol/L citric acid-0.1 mol/L Na_2_HPO_4_ buffers at pH 3.0–8.5 at 4°C. The residual *β*-mannanase activity was immediately measured after treatment by the DNS procedure.

### 2.8. The Effect of Metal Ions on the Activity of *β*-Mannanase

In order to examine the effects of metal ions on the activity of *β*-mannanase, the enzyme was incubated for 30 min at 4°C in the presence of various 1.0 mmol/L metal ions, CaCl_2_·2H_2_O, ZnCl_2_, FeCl_3_, PbCl_2_·2H_2_O, MnCl_2_·4H_2_O, MgCl_2_·6H_2_O, KCl, CuCl_2_·2H_2_O, AlCl_3_, BaCl_2_, and NH_4_Cl. The residual *β*-mannanase activity was measured at a specific condition and that of the treatment in the absence of additives as a control.

### 2.9. Substrate Specificity and Kinetic Parameters

Various glycans, such as Konjac glucomannan [23], locust bean gum from* Ceatonia siliqua* seeds (Sigma, G0753), carob galactomannan (Megazyme, P-GALML), guar galactomannan (Megazyme, P-GGMMV), ivory nut mannan (Megazyme, P-MANIV), 1,4-beta-D-mannan (Megazyme, P-MANCB), wheat arabinoxylan (Megazyme, P-120601a), beechwood xylan (Megazyme, P-141101a), and carboxymethyl cellulose (Megazyme, P-CMC4M) were examined. In brief, 0.2% (w/v) glycans were incubated with *β*-mannanase at 65°C for 10 min in 0.05 mol/L citric acid-0.1 mol/L Na_2_HPO_4_ buffer at pH 6.0, and the reducing sugars were measured by DNS. The Michaelis-Menten kinetic parameters, *V*_max_ and *K*_*m*_, were calculated for *β*-mannanase. The assays of the purified enzyme were carried out by the standard DNS procedure, as described above, using 1–5 mg/mL locust bean gum and 0.5–2.5 mg/mL Konjac glucomannan as substrates. The kinetic constants were determined from the Michaelis-Menten equation by directly inputting the initial rates from Lineweaver-Burk plots or the nonlinear regression [24].

### 2.10. Statistical Analysis

Each *β*-mannanase activity experiment was performed in triplicate and expressed as mean ± SD (standard deviation). The statistical analyses were performed with SPSS 15.0 (SPSS Inc., Chicago IL, USA). One-way or two-way analysis of variance (ANOVA) was used to compare various treatment groups.

## 3. Results and Discussion

### 3.1. Screening of the High *β*-Mannanase Activity Producing Strain

**Figure 1:**
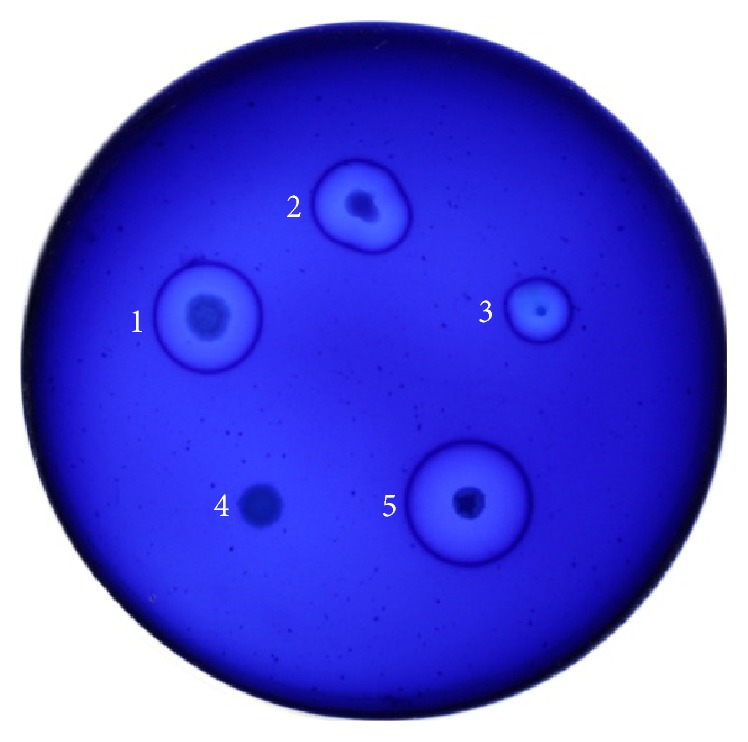
Clear halos produced by control and active colonies with *β*-mannanase activity 1, BE-78; 2, BE-46; 3, BE-83; 4, BE-23 (negative control); 5, BE-91.

Four bacteria were stochastically selected for the *β*-mannanase activity assay.  Figure 1 exhibited the halos produced on the screening plate.  Table 1 summarized the *β*-mannanase activity of the four bacteria (strain BE-23 without *β*-mannanase activity was used as a negative control). Strain BE-91 fermented for 9 h exhibited the highest activity, up to 273.7 IU/mL. Wild-type* B. subtilis* MA139 yielded a maximum *β*-mannanase activity of 170 IU/mL after 3 days of fermentation, and the maximum enzyme activity of* B. subtilis* TJ-102 was 205.3 IU/mL at 38 h [25, 26]. Notably, BE-91 secreted *β*-mannanase with higher activity in shorter time.

### 3.2. Classification of* B. subtilis* BE-91

**Figure 2:**
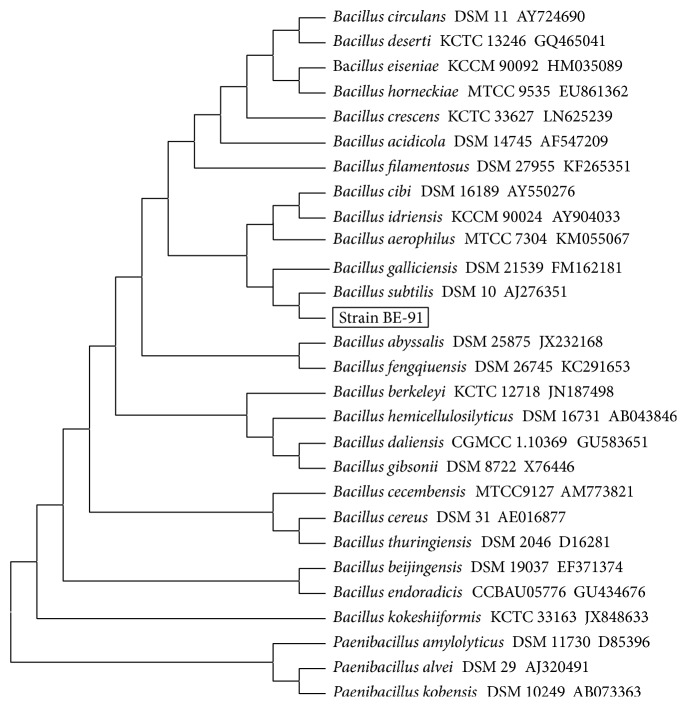
Phylogenetic tree based on 16S rDNA sequences of strain BE-91 and other bacteria by Mega 6.0 using neighbor-joining analysis with 1000 bootstrap replicates.

The 1,508 bp sequence of 16S rDNA of strain BE-91 was analyzed by a phylogenic tree (Figure 2). The homology between BE-91 16S rDNA (gi 260159552) and* B. subtilis* 16S rDNA (gi 530330588 and gi 341831474) was 99%. It was confirmed that the similarity of* B. subtilis* type strains about 16S rRNA gene sequence is higher than 98% [27, 28]. We also obtained ≥98% similarity to 16S rRNA gene sequences of* B. subtilis* isolates.

### 3.3. Isolation and Purification of *β*-Mannanase

**Figure 3:**
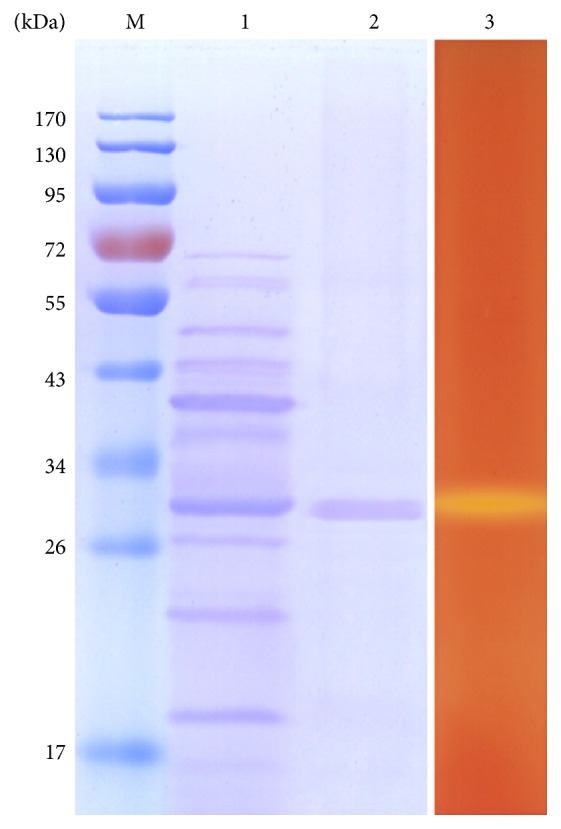
SDS-PAGE analysis of *β*-mannanase. Lane M: protein molecular weight standard; Lane 1: culture broth; Lane 2: purified *β*-mannanase; Lane 3: zymogram of purified *β*-mannanase.

2,000 mL of fermentation liquor was purified by ultrafiltration and chromatography. Specific activity, recovery, and multiple purifications at each step were summarized in Table 2. The recovery of *β*-mannanase in* B. subtilis* BE-91 exceeded 66.0%; multiple purifications achieved 32.9-fold pure *β*-mannanase activity, and the specific activity of the purified enzyme reached 79,859.2 IU/mg. The purified *β*-mannanase was shown to be homogeneous judged by SDS-PAGE analysis (Figure 3). Compared with the previous separation and purification methods [29, 30], the two-step method has the advantages of high efficiency, high yield, and easy operation.

### 3.4. Apparent Molecular Weight of *β*-Mannanase

The apparent molecular weight of *β*-mannanase was 28.2 kDa (Figure 3), lower than those of the most known *β*-mannanases from* Bacillus* spp. (*Bacillus licheniformis *THCM 3.1, 40 kDa;* B. subtilis* WY34, 39.6 kDa;* B. subtilis* Z-2, 38 kDa;* Bacillus circulans* CGMCC1554, 32 kDa) [28, 31–34]. Similarly, the molecular weights of *β*-mannanases from* Penicillium occitanis* Po16 and* Bacillus halodurans* PPKS-2 were 22 and 18 kDa, respectively [30, 31]. Due to low molecular weights, these enzymes may rapidly penetrate the lignocellulose systems and depolymerize the mannans more efficiently [35].

### 3.5. Optimal Temperature and Thermostability of *β*-Mannanase

**Figure 4:**
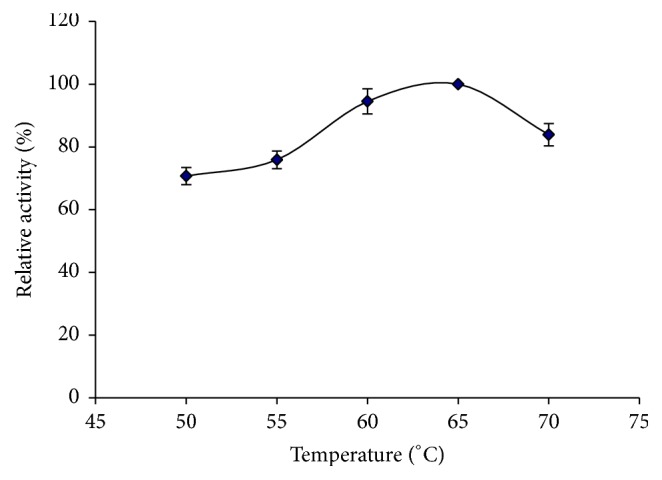
Optimum temperature curve of *β*-mannanase.

**Figure 5:**
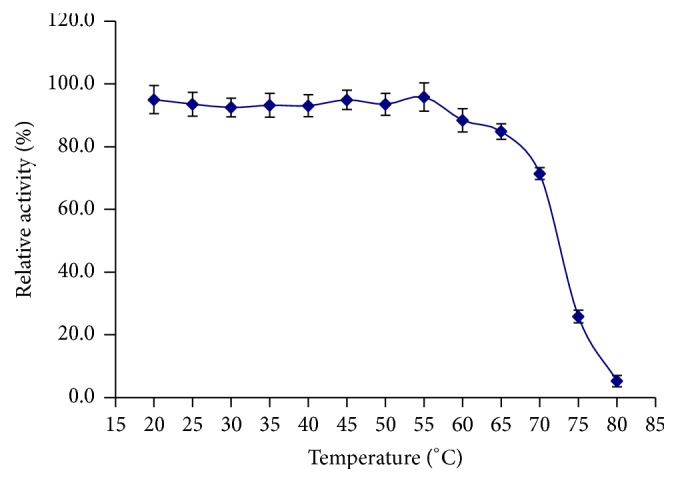
Thermal stability curve of *β*-mannanase.

The purified *β*-mannanase was maximally active at 65°C (Figure 4) and remained more than 80% active at 70°C (Figure 5). Compared with the optimal temperatures obtained for other *β*-mannanases (40°C for* Penicillium occitanis* Pol6; 50°C for both* Bacillus circulans *TN-31 and* B. subtilis *B36; 60°C for* Paenibacillus* sp. DZ3) [29, 31, 36], *β*-mannanase of BE-91 showed a pronounced activity at higher temperatures. As compared to the thermostability of the *β*-mannanase from wild-type* B. subtilis* BCC41051 (60°C for 30 min) [37], this *β*-mannanase retains 80% residual activity after incubation at 20–70°C for 30 min, indicating enhanced thermostability.

### 3.6. Optimal pH and Stability of *β*-Mannanase

**Figure 6:**
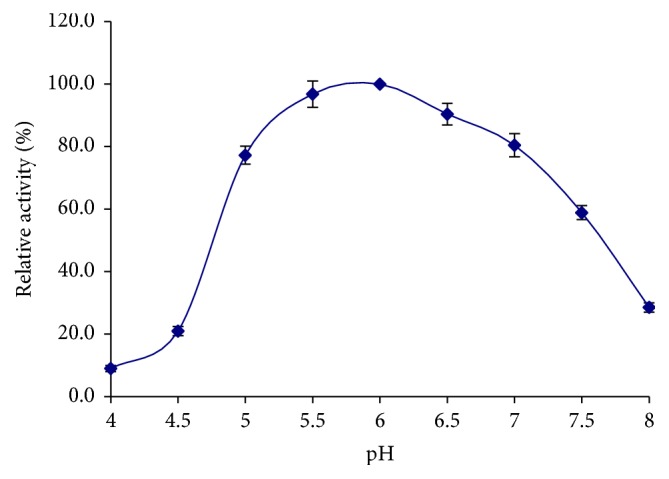
Optimum pH curve of *β*-mannanase.

**Figure 7:**
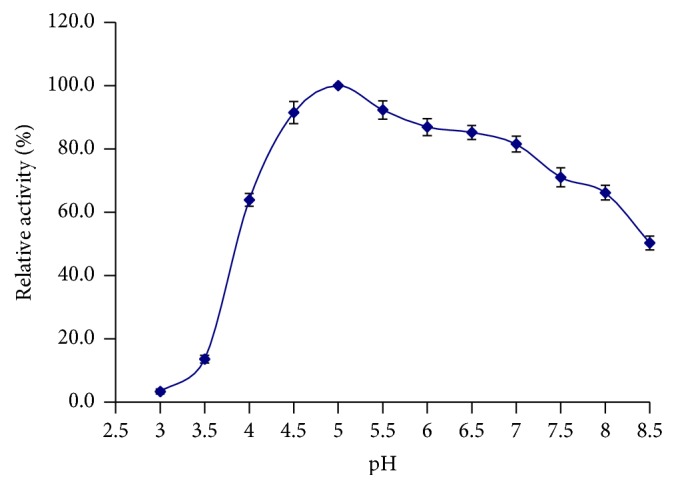
pH stability curve of *β*-mannanase.

The optimal pH and the stability of BE-91 *β*-mannanase were measured at various pHs. The optimum enzyme activity was obtained at pH 6.0 (Figure 6), and more than 80% maximal activity was retained at pH 4.5–7.0 (Figure 7). Interestingly, the optimal pH of BE-91 *β*-mannanase was the same as that of* B. subtilis* MA139 (pH 6.0), an enzyme that can potentially be used as a feed additive for monogastric animals [25]. At pH < 4.0, the *β*-mannanase activity was negligible, retaining <80% of its maximal value obtained after incubation at pH > 7.5, 4°C for 30 min. A relatively broad zone of optimum activity was observed. Therefore, BE-91 *β*-mannanase can be considered a weakly acidic and neutral enzyme, thereby rendering suitability for animal feed industry [38].

### 3.7. The Effect of Metal Ions on *β*-Mannanase Stability

The effect of a variety of metal ions on *β*-mannanase activity was measured (Table 3). The highest induction was achieved with Mn^2+^, which showed 168% baseline activity, followed by Al^3+^, Ca^2+^, Cu^2+^, Zn^2+^, Mg^2+^, and NH_4_^+^, respectively. K^+^ and Fe^3+^ had no obvious effects on *β*-mannanase activity in these conditions. Ba^2+^ and Pb^2+^ greatly inhibited the enzyme activity to a final rate of 83% and 74%, respectively. This suggests that BE-91 *β*-mannanase should not be contaminated by Ba^2+^ and Pb^2+^.

### 3.8. Kinetic Parameters

The purified enzyme hydrolyzed Konjac glucomannan but only slightly hydrolyzed ivory nut mannan, guar galactomannan, and 1,4-beta-D-mannan. Wheat arabinoxylan, beechwood xylan, and CMC were barely hydrolyzed, as shown in Table 4. This *β*-mannanase exhibited the highest activity with Konjac glucomannan, enriched in glucose units. This finding suggests that *β*-mannanase of BE-91 preferentially hydrolyzes the *β*-1,4-linkage of the glucosylated mannan backbone.

*K*_*m*_ and *V*_max_ values of this *β*-mannanase estimated by the Lineweaver-Burk plot were 7.14 mg/mL and 107.5 *μ*mol/min/mL, respectively, for locust bean gum, versus 1.749 mg/mL and 33.45 *μ*mol/min/mL for Konjac glucomannan, respectively. These results displayed higher affinity of *β*-mannanase towards natural Konjac glucomannan (*V*_max_/*K*_*m*_, 19.1 *μ*mol/min/mg) than the locust bean gum (*V*_max_/*K*_*m*_, 15.0 *μ*mol/min/mg), similar to the values obtained for* Penicillium pinophilum* C1 and* Penicillium freii* F63, hence constituting it as an adequate candidate in food industry for the production of oligosaccharides [17, 18, 39].

## 4. Conclusion

*B. subtilis* bacteria are abundant, moderately stable, and mostly nonpathogenic microorganisms. Our results indicated that* B. subtilis* BE-91 could be considered a prominent candidate for the production of extracellular *β*-mannanase. In addition, this study developed an advanced purification approach, “two-step method,” with high efficiency, high yield, and easy operation. Furthermore, the *β*-mannanase purified from BE-91 was extremely stable at relatively high temperatures and various weak acidic or neutral pHs. Finally, the enzyme showed a higher affinity towards natural Konjac glucomannan, a major functional food material. Therefore, this *β*-mannanase, purified and characterized from* B. subtilis* BE-91 for the first time, is suitable for inflammatory diseases.

## Figures and Tables

**Table 1:** *β*-mannanase activities of five bacteria.

Bacterium number	Activity (IU/mL)^a^	Specific activity (IU/mg)^a^
BE-23	0	0
BE-78	191.5 ± 4.5	879.8 ± 13.2
BE-46	83.2 ± 2.1	311.6 ± 9.4
BE-83	70.7 ± 1.6	119.7 ± 25.5
BE-91	273.7 ± 6.5	2,319.2 ± 26.3

^a^Data are mean ± SD, *n* = 3.

**Table 2:** Purification of *β*-mannanase by ultrafiltration and gel chromatography.

Purification step	Total activity (IU)	Total protein (mg)	Specific activity (IU/mg)	Recovery (%)	Purification multiple (fold)
Fermentation liquor	429,650.8	176.7	2,431.4	100	1
Ultrafiltration	328,317.4	8.6	38,070.2	76.4	15.6
Gel chromatography	283,500.2	3.6	79,859.2	66.0	32.9

**Table 3:** Effects of different metal ions (1 mmol/L) on *β*-mannanase activity.

Metal ions	Relative activity (%)^a^
Blank	100
K^+^	99 ± 3.2
NH_4_^+^	103 ± 2.7
Ca^2+^	117 ± 3.6
Zn^2+^	115 ± 2.9
Mn^2+^	168 ± 4.5
Cu^2+^	116 ± 2.1
Mg^2+^	107 ± 2.8
Ba^2+^	83 ± 3.1
Pb^2+^	74 ± 2.9
Fe^3+^	99 ± 3.6
Al^3+^	121 ± 4.3

^a^Data are mean ± SD, *n* = 3.

**Table 4:** Hydrolytic activity of the purified enzyme on different polysaccharides.

Substrate (0.5%, w/v)	Relative activity (%)^a^
Konjac glucomannan	100
Locust bean gum	88.15 ± 1.8
Carob galactomannan	91.85 ± 1.7
Guar galactomannan	35.70 ± 0.6
Ivory nut mannan	32.74 ± 0.3
1,4-Beta-D-mannan	46.22 ± 0.4
Wheat arabinoxylan	0
Beechwood xylan	0
Carboxymethyl cellulose	0

Assays were carried out at 65°C at pH 6.0 for 10 min in 0.05 mol/L citric acid-0.1 mol/L Na_2_HPO_4_ buffer.

^a^Data are mean ± SD, *n* = 3.
